# Potassium-Selective Solid-Contact Electrode with High-Capacitance Hydrous Iridium Dioxide in the Transduction Layer

**DOI:** 10.3390/membranes11040259

**Published:** 2021-04-04

**Authors:** Nikola Lenar, Robert Piech, Jan Wyrwa, Beata Paczosa-Bator

**Affiliations:** Faculty of Materials Science and Ceramics, AGH University of Science and Technology, Mickiewicza 30, PL-30059 Krakow, Poland; nlenar@agh.edu.pl (N.L.); rpiech@agh.edu.pl (R.P.); jwyrwa@uci.agh.edu.pl (J.W.)

**Keywords:** hydrous iridium dioxide, high electrical capacity, solid-contact layer, ion-selective electrodes, potassium determination

## Abstract

This work presents new material for solid-contact layers—hydrous iridium dioxide IrO_2_·2H_2_O, characterized by high electrical capacitance value, evaluated using chronopotentiometry (1.22 mF) and electrochemical impedance spectroscopy (1.57 mF). The remarkable electrical parameters of layers resulted in great analytical parameters of IrO_2_·2H_2_O-contacted potassium-selective electrodes. Various parameters of ion-selective electrodes were examined in the scope of this work using a potentiometry method including: linear range, repeatability, stability of potentiometric response and sensitivity to varying measurement conditions. The analytical parameters obtained for solid-contact electrodes were compared with the ones obtained for coated disc electrodes to evaluate the influence of the iridium dioxide layer. The linear range of the IrO_2_·2H_2_O-contacted K^+^-selective electrodes covered concentrations of K^+^ ions from 10^−6^ to 10^−1^ M and the potential stability was estimated at 0.097 mV/h. The IrO_2_·2H_2_O-contacted electrodes turned out to be insensitive to varying light exposure and changes in the pH values of measured solutions (in the pH range of 2 to 10.5). A water layer test proved that, contrary to the coated disc electrode, the substantial water film is not formed between the ion-selective membrane and iridium dioxide layer.

## 1. Introduction

The transition from conventional ion-selective electrodes into all-solid-state electrodes created a wide range of applications and promises to simplify electrode construction. As consequence, the internal solution had to be replaced by a solid ion-to-electron transduction layer to ensure the stable potentiometric response of electrodes [1,2]. Implementing and recognizing new functional materials for solid-contact layers in ion-selective electrodes is one of the most active topics in the scope of the potentiometry method. Amongst the materials applied till this day, next to the conducting polymers and carbon nanomaterials, we can distinguish the group of metal oxide nanomaterials. Metal oxide nanomaterials exhibit ion-to-electron transduction resulting from both the high surface area guaranteed by the nanometric size of the oxide’s particles, and high redox activity [3]. The first oxides to be implemented into ion-selective electrode construction were ZnO [4] and CuO [5]; later on, Qin et al. introduced MoO_2_ microspheres [6], Yang et al. introduced MnO_2_ nanosheets [7], and our, group led by Paczosa-Bator, introduced ruthenium dioxide nanoparticles [8,9].

This work focuses on introducing a new metal oxide into K^+^-selective electrode construction—hydrous iridium oxide. Replacing the internal solution in conventional potassium-selective electrodes with an iridium dioxide mediation layer allowed not only to simplify and miniaturize the construction of electrodes but also, thanks to the high electrical capacity of IrO_2_, to improve the stability of potentiometric response. 

Iridium dioxide belongs to the group of transition metal oxides classified as pseudo capacitors, characterized by high theoretical specific capacitance resulting from the faradaic charge transfer process [10]. The pseudocapacitance of IrO_2_ implies that reversible redox reactions occur at or near the material’s surface when contacting the electrolyte [11]. Iridium dioxide may be used as a sensing material itself as it exhibits pH-sensing properties and was repeatedly used for this purpose [12]. Iridium dioxide is also applied in catalytic applications due to its outstanding electrocatalytic properties for anodic reactions [13]. In this work, we present a different possibility of applying hydrous IrO_2_ in the field of electrochemical analysis—as a mediation layer in solid-contact ion selective electrodes. 

In the previous works published by our group [8,9,14], the other transition metal oxide, ruthenium dioxide, was discussed. Similarly to RuO_2_, IrO_2_ crystallizes in the rutile structure [12] and exists in two forms—hydrous and anhydrous. The difference between hydrous and anhydrous metal oxide lays in the porosity and the presence of the water layer. The water film in between the oxide’s grains allows protons and electrons to be transported through the oxide’s layer. On the other hand, the increased porosity of the hydrous structure ensures a shorter diffusion distance [11,15,16,17]. Taking into consideration the mentioned features and differences between both types of oxides, it can be concluded that hydrous oxide exhibits more favorable characteristics in the context of designing potentiometric sensors. In the case of ruthenium, the hydrous form turned out to be more favorable for ion-selective electrodes than anhydrous, as presented in [14]; therefore, when applying iridium oxide as a mediation layer, its hydrous form was chosen. 

This work contains both the characterization of hydrous iridium dioxide as a material for the mediation layer and the characterization of ion-selective electrodes with the IrO_2_·2H_2_O layer. 

## 2. Materials and Methods

### 2.1. Chemicals 

The chemicals used for the purpose of this study can be divided into those for electrode preparation and those for standard solution preparation. Solid-contact ion-selective electrodes consist of two layers applied onto the surface of the electrode (in this case, glassy carbon disc electrode)—the mediation layer and the ion-selective membrane. As the mediation layer, we implemented iridium dioxide dihydrate (Alfa Aesar) dispersed in DMF (Sigma-Aldrich, St. Louis, MO, USA). The potassium-selective membrane cocktail consists of the following components: potassium ionophore I (Valinomycin), lipophilic salt-potassium tetrakis(4-chlorophenyl)borate (KTpClPB), 2-nitrophenyl octyl ether (o-NPOE) and poly(vinyl chloride) (PVC), all purchased from Sigma-Aldrich. All the components were dissolved in tetrahydrofuran THF (Sigma-Aldrich). 

Aqueous standard solutions of K^+^ concentration range from 10^−7^ to 10^−1^ M used for the calibration and electrochemical measurements were prepared by dissolving 1M KCl solution (potassium chloride purchased from POCH, Gliwice, Poland). Standard NaCl solution used during the water layer test was prepared based on the sodium chloride purchased from POCH, as well as sodium hydroxide and hydrochloric acid used to adjust the pH value of KCl solution during the pH sensitivity test. 

For the aqueous solution preparation, distilled and deionized water was used. All the chemicals were applied as obtained, without any further purification.

### 2.2. Electrodes Preparation

For the purpose of this work, three items of IrO_2_·2H_2_O-contacted K^+^-selective electrodes and three items of coated-disc K^+^-selective electrodes were prepared. The group of GC/K^+^-ISM electrodes was tested as a control to evaluate the influence of the iridium dioxide layer on the ion-selective electrode’s properties. Solid-contact electrodes consist of a mediation layer and ion-selective membrane. Material for the mediation layer was prepared by dispersing 7 mg of iridium dioxide in 1 mL of dimethylformamide (DMF) using an ultrasonic bath. A potassium-selective membrane was obtained by dissolving membrane components: ionophore I—2.81 μg, lipophilic salt KTpClPB—0.64 μg, plasticizer o-NPOE—167.41 μg, and PVC—84.25 μg in 2 mL of tetrahydrofuran (THF).

Before casting, glassy carbon disc (GCD) electrodes were polished on alumina slurries (0.3 and 0.05 μm) and cleaned ultrasonically in water (2 min) and methanol (1 min). As soon as the surface of the electrodes was prepared, 15 μL of IrO_2_·2H_2_O dispersed in DMF (of 7 mg/mL concentration) was dropped onto the electrodes (three items) and dried at an elevated temperature until solvent evaporation. Solid particles of iridium dioxide were subsequently covered with 60 μL of ion-selective membrane. At the same time, another three GC electrodes were covered with the ion-selective membrane cocktail. All six electrodes were left to dry at room temperature and, later on, conditioned in 0.01 M KCl solution before conducting the series of measurements and tests. The proposed preparation method is easy and as fast as several minutes to carry out. 

### 2.3. Methods

The methods applied for the examination of the material for the mediation layer included scanning electron microscopy and electrochemical techniques such as chronopotentiometry and electrochemical impedance spectroscopy (EIS). The electrodes were tested using three electrochemical techniques: EIS, chronopotentiometry, and potentiometry.

An LEO 1530 scanning electron microscope (Carl Zeiss, Oberkochen, Germany) was used for examination of the hydrous iridium dioxide layer. 

Electrochemical techniques were used for evaluating the electrical parameters of layers including capacitance and resistance. For chronopotentiometric and EIS measurements, AUTOLAB PGSTAT302N analyzer with an FRA module (Eco Chemie, Utrecht, The Netherlands) and data stimulating and analyzing software (NOVA 2.1) were used. Both measurements were conducted in the 3-electrode cell consisting of Ag/AgCl reference electrode (ΩMetrohm, Herisau, Switzerland, type 6.0733.100 with 3 M KCl solution), auxiliary glassy carbon electrode and working electrode—glassy carbon disc electrode with the studied layer (or a layer covered with ion-selective membrane). The cell was filled with 0.01 M KCl solution when carrying out both chronopotentiometric and EIS measurements. 

A potentiometry method was used for evaluating the analytical parameters of potassium-selective electrodes, including: repeatability, reproducibility, linear range, potential stability, and light and pH sensitivity. The 16-channel potentiometer (Lawson Labs, Inc., Malvern, PA, USA) was used for electromotive force (EMF) registration. All measurements were conducted against the reference Ag/AgCl electrode (ΩMetrohm, Switzerland, type 6.0733.100) and in the presence of the auxiliary electrode—a platinum wire. Calibration curves were recorded using 10^−7^ to 10^−1^ M K^+^ ions standard solutions and stability and sensitivity measurements were conducted in 10^−2^ M K^+^ ions solution. 

## 3. Results and Discussion

### 3.1. Layer’s Morphology

**Figure 1:**
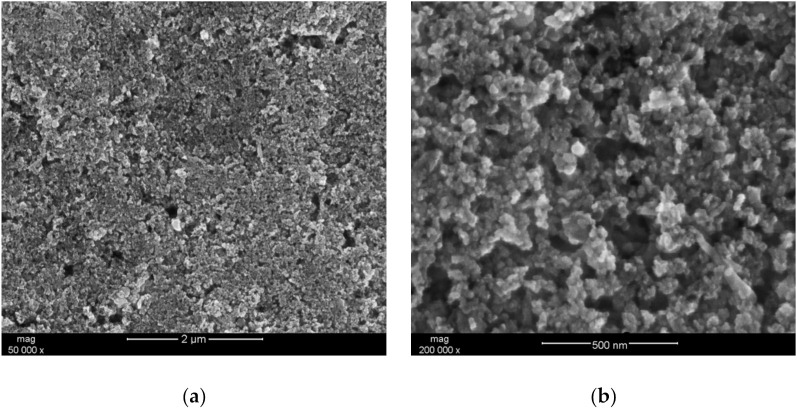
SEM (Scanning Electron Microscope) scan depicting the morphology of hydrous iridium dioxide layer. (**a**) 50,000 × magnitude; (**b**) 200,000 × magnitude.

In order to examine the microstructure of the hydrous iridium dioxide layer, a droplet (~50 μL) of the dioxide solution in DMF was casted onto a platinum pad and dried at an elevated temperature (equivalently as it was practiced with electrodes). The prepared sample was placed into the sample chamber of the scanning electron microscope and analyzed using different magnitudes. The obtained images are presented in Figure 1a,b for 50,000 × magnitude and for 200,000 × magnitude, respectively. The scans depict nanometric particles of IrO_2_·2H_2_O distributed evenly throughout the sample. The small size of the oxide’s grains and visible roughness and porosity suggest that the obtained layer is characterized by a high surface area, which is considered to be a favorable feature in the context of designing mediation layers for ion-selective electrodes. Since the ion-to-electron exchange processes take place in the interface between the layer and ion-selective membrane, the high surface area of the solid contact layer facilitates those processes, improving, simultaneously, the electrical capacity of an electrode [3].

Taking all of the above into consideration, the electrical parameters of layers and electrodes were tested using various electrochemical techniques to confirm the beneficial influence of the high-surface-area iridium dioxide layer on the ISE parameters. 

### 3.2. Electrical Capacitance 

#### 3.2.1. EIS

Electrochemical impedance spectroscopy was incorporated into the study to evaluate the charge storage properties of iridium dioxide. The ability of the layer to store the charge determined by the double-layer/redox capacitance parameter enables to sustain the equilibrium in the presence of external disturbances and protects the electrode from the impact of the current flow during the potentiometric measurement. It is therefore important to examine the electrical capacity of the layer before applying it as a solid-contact layer in ion-selective electrodes. 

**Figure 2:**
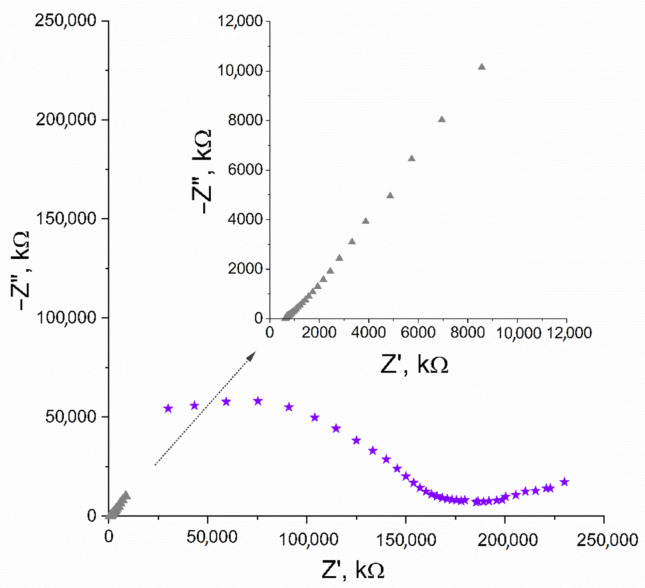
Nyquist plot recorded for IrO_2_·2H_2_O solid-contact layer (▲) and GC/IrO_2_·2H_2_O/K^+^-ISM electrode (★) in the 10^−2^ M K^+^ ion solution. Inset: enlarged Nyquist plot of IrO_2_·2H_2_O solid-contact layer (▲).

In EIS measurement, the capacitance (C) parameter can be obtained from the imaginary part of the impedance value (Z”) recorded for low frequencies (ƒ) using the C = 1/(2πƒZ”) equation. The first and last applied frequency was 100 kHz and 0.01 Hz, respectively, and the amplitude set versus open circuit potential was 0 V. The capacitance value calculated for the lowest frequency ƒ = 0.01 V equaled 1.57 mF. The Nyquist plot, where the imaginary part of impedance is plotted in the function of real impedance, is presented in Figure 2.

The Nyquist plot of GC/IrO_2_·2H_2_O/K^+^-ISM electrode was obtained using equivalent parameters and presented in Figure 2, together with the plot obtained for the iridium dioxide layer itself. The capacitance parameter calculated for the studied electrode was 0.92 mF.

#### 3.2.2. Chronopotentiometry

**Figure 3:**
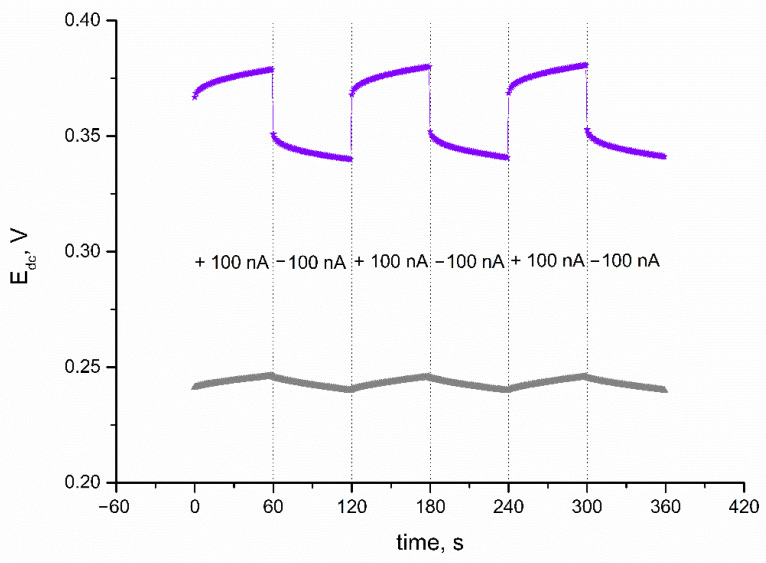
Chronopotentiograms recorded for IrO_2_·2H_2_O solid-contact layer (▲) and GC/IrO_2_·2H_2_O/K^+^-ISM electrode (★) with the current flow of 100 nA.

A chronopotentiometry method was also applied to evaluate the electrical capacitance of IrO_2_·2H_2_O layer, and subsequently, the electrical parameters of IrO_2_·2H_2_O-contacted electrodes. The technique was programmed so the 100 nA current (I) passed through the cell during the measurement. The program lasted 360 s, and every 60 s, the current sign changed (starting with positive sign) and the potential (E_dc_) was recorded with time (t). First, the CG electrode was casted with iridium dioxide in DMF to examine the electrical properties of the layer itself. The electrical capacity is characterized by the capacitance parameter, which can be calculated using the C = I(dt/dE_dc_) equation presented by Bobacka et al. in [18]. The electrical capacitance was calculated for the linear parts of recorded chronopotentiogram (presented in Figure 3). As expected, because of the nanometric size of the oxide’s particles and oxide’s ability to participate in redox processes, the electrical capacitance of the solid-contact layer turned out to be significantly high—1.22 ± 0.03 mF. Another electrical parameter that characterizes functional materials applied in electrochemical sensors is resistance. The resistance parameter is calculated based on the value of potential jump (△E_dc_) using the R_total_ = △E_dc_/2I equation. For the examined material, the resistance value was 2.20 ± 0.08 kΩ.

### 3.3. Electrical Parameters of Electrodes

Electrical parameters of IrO_2_·2H_2_O-contacted electrodes were evaluated using the same program and same equations as for the iridium dioxide layer itself. The electrical parameters of the K^+^-selective electrode were calculated for the linear parts of the obtained chronopotentiogram (presented in Figure 3 together with the chronopotentiogram recorded for the layer itself). GC/IrO_2_·2H_2_O/K^+^-ISM electrodes exhibit an electrical capacitance of 0.93 ± 0.04 mF and resistance of 140 ± 1 kΩ. Additionally, for the solid-contact electrodes, the potential drift dE/dt was calculated. Since a high electrical capacity determines a stable potentiometric response, the potential drift was minor—107 ± 3 μV/s.

The electrical capacity of the solid-contact layers and solid-contact electrodes evaluated using chronopotentiometry and electrochemical impedance spectroscopy and characterized with the electrical capacity parameter was compared in the Table 1. 

When comparing the electrical capacitance parameter calculated for the solid-contact layer itself and the electrodes with the solid-contact layer, based on the data obtained from two different electrochemical techniques, it can be seen that the electrical capacity of the layer is higher without the presence of an ion-selective membrane. The results obtained using chronopotentiometry and EIS techniques for the IrO_2_·2H_2_O layer are consistent (0.92 and 0.93 mF, respectively), while in the case of IrO_2_·2H_2_O-contacted electrode, the electrical capacitance value obtained from the second technique (EIS—1.57 mF) is higher than for the first one (chronopotentiometry—1.22 mF). What should be emphasized here is that the electrical capacity of the layers and ready-to-use electrodes are competing to the electrodes based on similar (to hydrous iridium dioxide) materials. Hydrous ruthenium dioxide presented by our group in previous work as a mediation layer allowed to increase the electrical capacitance parameter to 1.07 mF. Those values (0.93 and 1.07 mF for IrO_2_·2H_2_O and RuO_2_·2H_2_O-contacted electrodes) are significantly higher than those obtained for other metal oxide micro and nanomaterials, e.g., MoO_2_ microspheres (0.086 mF) [6] or MnO_2_ nanosheets (0.029 mF) [7]. Higher values of electrical capacitance are attributed to nanocomposites consisting of more than one component, yet for the single component layer, hydrous iridium dioxide, together with ruthenium dioxide and colloid-imprinted mesoporous carbon (electrical capacity of CIMC-contacted electrode equal to 1.0 mF) [19] exhibit some of the highest electrical capacities. 

### 3.4. Potentiometric Response of K^+^-Selective Electrodes

**Figure 4:**
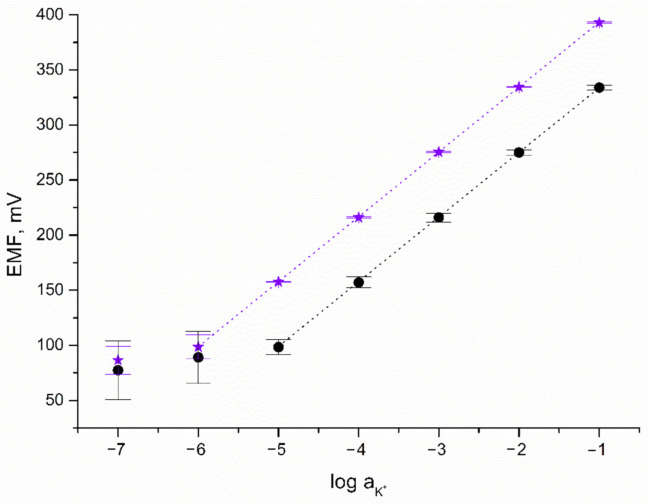
Potentiometric response of GC/IrO_2_·2H_2_O/K^+^-ISM (★) and GC/K^+^-ISM electrode (●) towards K^+^ ions.

The response towards potassium ions was tested during potentiometric measurements using KCl standard solutions of increasing the concentration of K^+^ ions (from 10^−7^ to 10^−1^ M). To examine the potentiometric response of the designed IrO_2_·2H_2_O-contacted electrodes, calibration curves were recorded over nine days (after 24, 144, and 216 h of electrode-conditioning in 0.01 M K^+^ ion solution). For comparison, the potentiometric response of CG/K^+^-ISM electrode was also recorded. Calibration curves of both solid-contact and coated disc electrodes together with error bars depicting the repeatability of the potentiometric response over nine days are presented in Figure 4.

As can be seen, the linear range of solid-contact electrodes turned out to be wider contrary to the linear range observed for the coated disc electrode. Near-Nernstian response (with a calibration curve slope of 59.29 and 58.99 mV/pK for GC/IrO_2_·2H_2_O/K^+^-ISM and GC/K^+^-ISM electrode, respectively) was observed for K^+^ ion concentrations from 10^−6^ to 10^−1^ M for solid-contact and 10^−5^ to 10^−1^ M for coated disc electrode. This proves that the applied iridium dioxide mediation layer beneficially affects the potentiometric response of ion-selective electrodes by expanding their linear range. 

The detection limit of GC/IrO_2_·2H_2_O/K^+^-ISM electrode was equal to 10^−6.2^ M, which was higher than that of MoO_2_—(10^−5.5^) [6], MnO_2_—(10^−5.2^) [7], and CIMC-contacted K^+^-selective electrodes (10^−5.6^ M) [19] and comparable to the LOD obtained for RuO_2_-contacted K^+^-ISE (10^−6.5^) [8]. 

Based on the standard deviation values calculated for potential recorded over nine days for one electrode representing each group, the repeatability of both GC/IrO_2_·2H_2_O/K^+^-ISM and GC/K^+^-ISM electrodes was evaluated and compared. The standard deviation values are presented in Figure 4 as error bars. For GC/IrO_2_·2H_2_O/K^+^-ISM electrode SD values equal to not more than 0.5 mV for a K^+^ ion concentration between 10^−1^ and 10^−5^ M while for the same concentration range, SD values for the GC/K^+^-ISM electrode are up to 7 mV. For lower concentrations of potassium ions, standard deviation values are more significant and for 10^−7^ M concentration up to 13 mV for solid-contact and up to 27 mV for coated disc electrode. As presented, the SD values are twice as high for electrodes without a mediation layer than for IrO_2_·2H_2_O-contacted electrode, which proves that solid-contact electrodes are characterized by greater repeatability. 

Another analytical parameter characterizing the potentiometric response of ion-selective electrodes is reproducibility, indicating the convergence of electrodes’ potentiometric response within one group of electrodes. The reproducibility parameter is evaluated based on the standard deviation value of electrodes’ standard potential E_0_. After 24 h of conditioning for the group of IrO_2_·2H_2_O-contacted electrodes, the SD, calculated based on the results obtained for three items forming the group, was 5 mV, while for the group of coated disc electrodes, it was 19 mV (n = 3). As expected, the presence of a hydrous dioxide mediation layer beneficially affects the reproducibility of K^+^-selective electrodes’ response. 

### 3.5. Potential Stability

**Figure 5:**
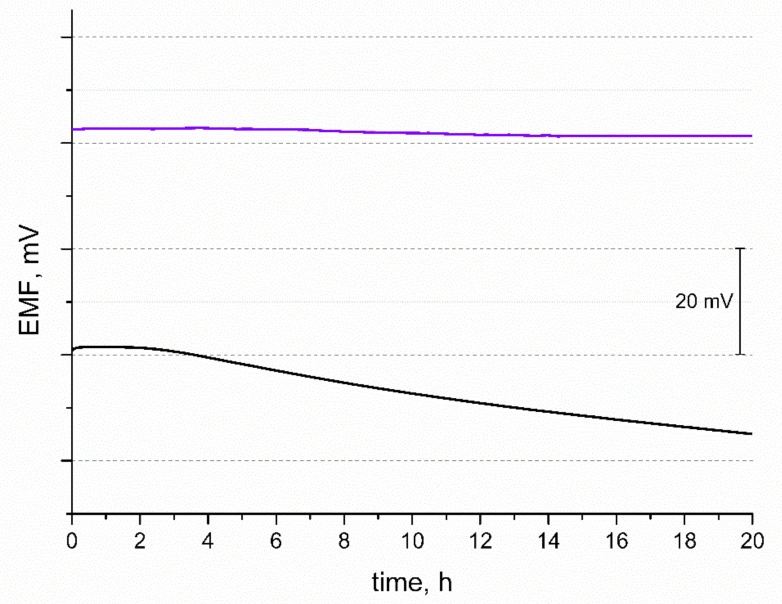
Stability of potentiometric response of GC/IrO_2_·2H_2_O/K^+^-ISM (purple line) and GC/K^+^-ISM electrode (black line) recorded over 20 h in 0.01 M KCl solution.

Another tested feature of the potentiometric response was potential stability. The EMF was recorded over 20 h in 10^−2^ M standard K^+^ ion solution and the obtained curves are presented in Figure 5. During the time of measurement, the potential drift of response was observed and calculated for both solid-contact and coated disc electrodes (for comparison). The potential stability parameter given by the △EMF/△t ratio was 0.063 mV/h and 0.83 mV/h for GC/IrO_2_·2H_2_O/K^+^-ISM and GC/K^+^-ISM electrode, respectively. The difference in the potential stability of both tested electrodes may be explained by the difference in their electrical capacity. Electrodes characterized by a greater electrical capacitance parameter tend to exhibit a more stable potentiometric response with time due to their insensitivity to the perturbations that may occur during the measurement, e.g., power fluctuations [18]. 

### 3.6. Light Test

**Figure 6:**
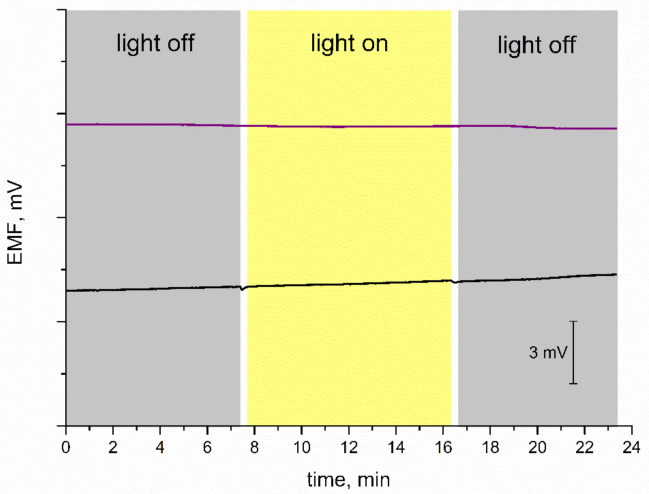
Light test: stability of potentiometric response of GC/IrO_2_·2H_2_O/K^+^-ISM (purple line) and GC/K^+^-ISM electrode (black line) recorded for varying light conditions in 0.01 M KCl solution.

Subsequently, the stability of the potentiometric response was tested for varying light conditions. Some materials used as mediation layers turned out to be sensitive to changes in the light intensity; therefore, it is important to evaluate the stability of EMF recorded for electrodes with new materials. For the purpose of this measurement, the light was off during the first few minutes of the test, then on for another several minutes, and off at the end, as presented in Figure 6. Since coated disc electrodes without a mediation layer are characterized as insensitive to light conditions, the curve recorded for the GC/K^+^-ISM electrode was presented next to the one obtained for IrO_2_·2H_2_O-contacted electrode. Both electrodes exhibit a stable potentiometric response with time regardless of the changing light intensity. The test confirmed that GC/IrO_2_·2H_2_O/K^+^-ISM are insensitive to the varying light conditions and that the iridium dioxide layer does not affect the potentiometric response in terms of light sensitivity. 

### 3.7. pH Sensitivity

**Figure 7:**
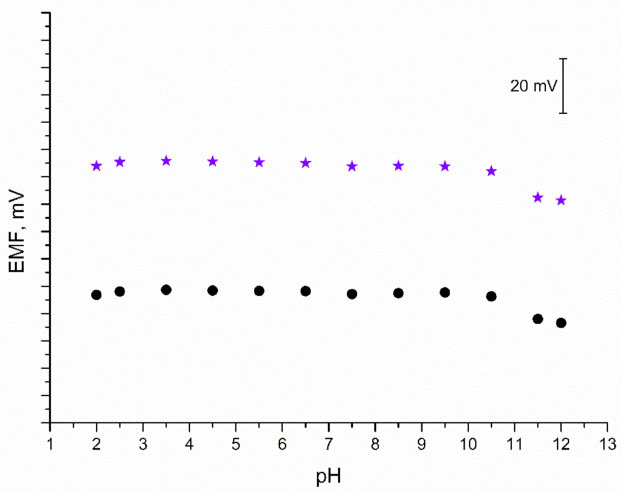
pH test: stability of potentiometric response of GC/IrO_2_·2H_2_O/K^+^-ISM (★) and GC/K^+^-ISM electrode (●) recorded in 0.01 M KCl solutions of varying pH value (2–12).

As mentioned in the Introduction section, iridium dioxide exhibits sensitivity towards hydrogen ions; therefore, it is important to confirm the insensitivity of IrO_2_·2H_2_O-contacted electrodes to a changing pH value. For the purpose of this test, a series of 0.01 M K^+^ ion solutions of varying pH values were prepared. Each solution was titrated either with 3M sodium hydroxide (to obtain solutions of pH from 6 to 12) or with 3M hydrochloric acid (to receive solutions of pH from 2 to 5). The EMF was recorded for each solution and the obtained results are presented in Figure 7. The test confirmed that both solid-contact and coated disc electrodes are insensitive to changes in pH between 2 and 10.5. In the extreme alkalic conditions (pH 11.5 and 12) the potentiometric response is disrupted; therefore, the designed electrodes cannot be applied for measurements in such conditions. 

### 3.8. Water Layer Test

One of the disadvantages resulting from removing the internal solution from ion-selective electrodes’ construction and replacing it with a solid-contact layer is the formation of a thin water film in the interface between the ion-selective membrane and electrode’s surface. This phenomenon affects both the performance and analytical properties of electrodes. A water layer makes it impossible for the membrane to adhere properly to the electrode and with time may cause its detachment, leading to mechanical failure. On the other hand, the presence of a water layer deteriorates the long-term potential stability of electrodes [3,20]. One of the approaches to eliminate the water layer is covering the electrode’s surface with a mediation layer that is in good contact with the polymeric membrane. In order to evaluate the ability of iridium dioxide to prevent the formation of an aqueous layer, the water layer test was conducted. 

**Figure 8:**
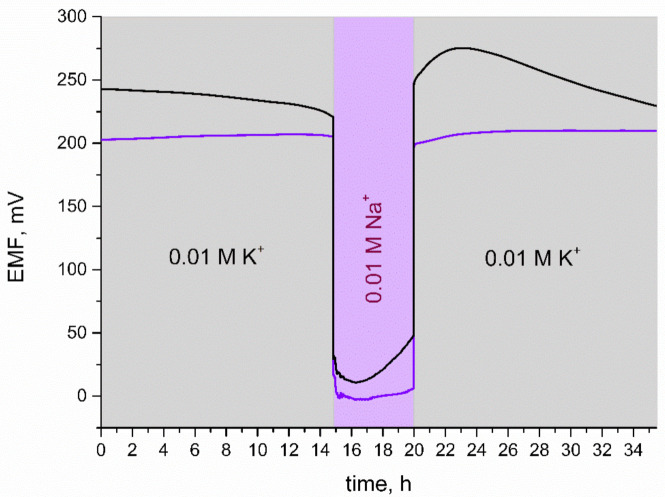
Water layer test: stability of potentiometric response of GC/IrO_2_·2H_2_O/K^+^-ISM (purple line) and GC/K^+^-ISM electrode (black line) recorded in 0.01 M KCl and 0.01 M NaCl solutions.

For the purpose of this test, 0.01 M KCl and 0.01 M NaCl solutions were used. As presented in Figure 8, for the first 20 h of measurement, electrodes were placed in a K^+^ ion solution and then the primary ion was changed into Na^+^ for several hours. In the last step, electrodes were contacted with K^+^ ions and the potential response was observed. 

When comparing the response of the solid-contact and coated disc electrode during the water layer test, it can be seen that GC/K^+^-ISM electrodes exhibit a substantial potential drift when exchanging K^+^ ions into Na^+^ ions in the analyzed solution. IrO_2_·2H_2_O-contacted electrodes, after being placed back into K^+^ ions solution, exhibited fast stabilization of EMF and potential drift was not observed. It can therefore be concluded that, contrary to coasted-disc electrodes, GC/IrO_2_·2H_2_O/K^+^-ISM electrodes do not contain a substantial aqueous layer.

## 4. Conclusions

Hydrous iridium dioxide was examined as a solid-contact layer for all-solid-state potentiometric sensors on the example of potassium selective electrodes. Taking into consideration all the results collected for IrO_2_·2H_2_O-contacted electrodes and the iridium dioxide layer itself, it can be concluded that this material is suitable for the mediation layer for ion-selective electrodes. 

The electrical capacity of the material was evaluated using chronopotentiometry and electrochemical impedance spectroscopy, and both techniques showed high capacitance values of 1.22 and 1.56 mF, respectively. The ability of the layer to store the charge, determined by the capacitance parameter, enables the sustained equilibrium of the electrodes’ response in the presence of external disturbances. The high capacity of solid-contact functional material resulted in the high electrical capacity of potassium-selective electrodes (0.93 mF), which led to the great stability of the potentiometric response given by a potential drift of 0.097 mV/h. 

The designed GC/IrO_2_·2H_2_O/K^+^-ISM electrodes may be applied for potassium-ion determination in the scope of K^+^ ion concentrations from 10^−6^ to 10^−1^, as, in this range, the near-Nernstian response (of 59.29 mV/dec) was observed. IrO_2_·2H_2_O-contacted electrodes exhibit a great repeatability of the potentiometric response in comparison with GC/K^+^-ISM electrode. 

The stability of the potentiometric response of electrodes with an iridium dioxide layer was tested in demanding conditions that may occur during potentiometric measurement including changes in the light intensity and pH value of the analyzed solutions. GC/IrO_2_·2H_2_O/K^+^-ISM electrodes turned out to be insensitive to light exposition and, regardless of iridium dioxide’s sensitivity to hydrogen ions, insensitive to changes to the pH value (between pH 2 and 10.5).

The conducted water layer test proved that iridium dioxide as a solid-contact layer successfully suppresses the formation of a water layer, protecting the electrode from delamination and deteriorating its long-term stability potential.

## Figures and Tables

**Table 1:** Electrical capacitance parameter obtained for IrO_2_·2H_2_O solid-contact layer and IrO_2_·2H_2_O-contacted electrode using two electrochemical techniques.

	Electrical Capacitance [mF]	
	Chronopotentiometry	EIS
IrO_2_·2H_2_O layer	1.22	1.57
IrO_2_·2H_2_O-contacted K^+^-selective electrodes	0.92	0.93

## Data Availability

Not applicable.
